# HERC Ubiquitin Ligases in Cancer

**DOI:** 10.3390/cancers12061653

**Published:** 2020-06-22

**Authors:** Joan Sala-Gaston, Arturo Martinez-Martinez, Leonardo Pedrazza, L. Francisco Lorenzo-Martín, Rubén Caloto, Xosé R. Bustelo, Francesc Ventura, Jose Luis Rosa

**Affiliations:** 1 Departament de Ciències Fisiològiques, Institut d’Investigació de Bellvitge (IDIBELL), Universitat de Barcelona, L’Hospitalet de Llobregat, 08907 Barcelona, Spain; joansala@ub.edu (J.S.-G.); arturomartinez@ub.edu (A.M.-M.); leopedrazza@gmail.com (L.P.); fventura@ub.edu (F.V.); 2 Centro de Investigación del Cáncer, Instituto de Biología Molecular y Celular del Cáncer and CIBERONC, Consejo Superior de Investigaciones Científicas (CSIC)-University of Salamanca, 37007 Salamanca, Spain; luis.lorenzomartin@epfl.ch (L.F.L.-M.); ruben.fdez.caloto@usal.es (R.C.); xbustelo@usal.es (X.R.B.)

**Keywords:** HECT, E3, oncogene, tumor suppressor, genome stability, p53, MAPK, RAF, ERK, p38

## Abstract

HERC proteins are ubiquitin E3 ligases of the HECT family. The HERC subfamily is composed of six members classified by size into large (HERC1 and HERC2) and small (HERC3–HERC6). HERC family ubiquitin ligases regulate important cellular processes, such as neurodevelopment, DNA damage response, cell proliferation, cell migration, and immune responses. Accumulating evidence also shows that this family plays critical roles in cancer. In this review, we provide an integrated view of the role of these ligases in cancer, highlighting their bivalent functions as either oncogenes or tumor suppressors, depending on the tumor type. We include a discussion of both the molecular mechanisms involved and the potential therapeutic strategies.

## 1. Introduction

**Figure 1:**
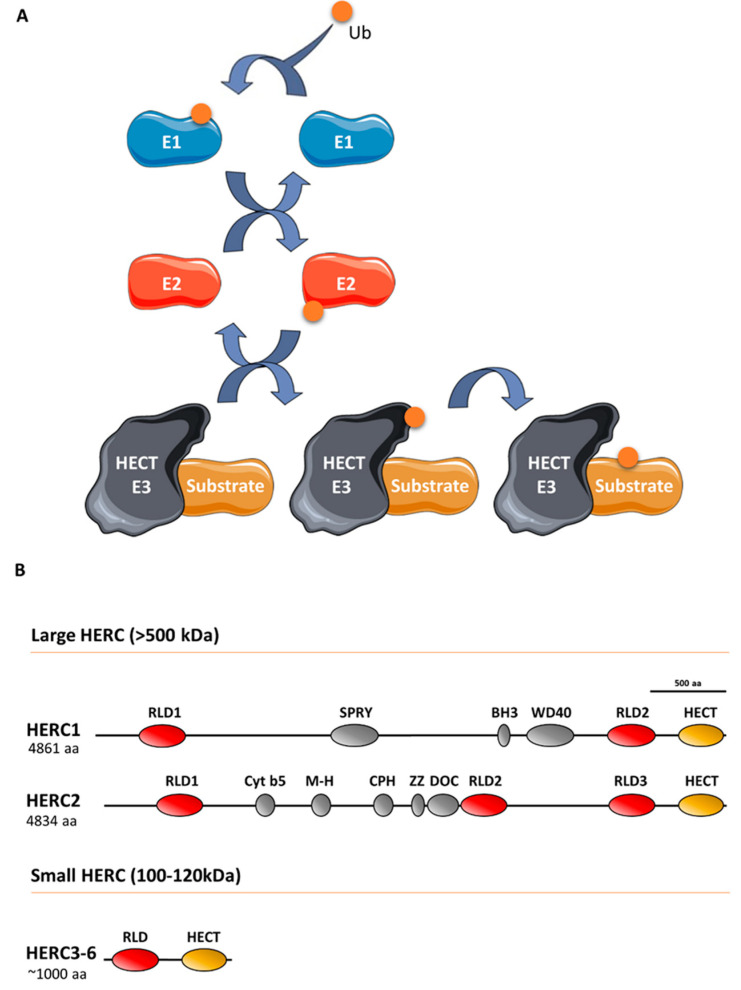
The ubiquitin-conjugating system in HERC E3 ligases: (**A**) Ubiquitin (Ub) is conjugated to a target substrate via a cascade that comprises an E1 activating enzyme, an E2 conjugating enzyme, and an E3 ligase enzyme. The HERC proteins belong to the HECT family of E3 ligases, which form a thioester bond with Ub via a conserved cysteine residue. Once formed, Ub is transferred to the substrate’s lysine residue (see text for details). Ub-like proteins, such as ISG15, are also covalently attached to the substrate protein via an E1/E2/E3 cascade; (**B**) structural features of large and small HERC proteins are also shown. HERC5 and HERC6 may also function as ISG15 E3 ligases.

Ubiquitin E3 ligases take part in protein ubiquitylation. These enzymes catalyze the last step of a cascade where ubiquitin is initially incorporated to a ubiquitin-activating enzyme (E1), which in turn is transferred to a ubiquitin-conjugating enzyme (E2), and finally, to a target protein through a process defined by a ubiquitin E3 ligase that interacts with the substrate protein (Figure 1). The ubiquitin-like proteins SUMO, NEDD8, and ISG15 are also covalently attached to the target protein via an E1/E2/E3 cascade. Specifically, the E3 ligases can be classified into three groups, of which one is homologous to the E6AP carboxyl terminus (HECT) protein. All HECT ligases have a catalytic domain in their carboxyl terminus that contains a conserved cysteine residue that is involved in forming a transiently thioester bond to ubiquitin before transferring it to the lysine residue of the substrate protein (Figure 1) [1]. HECT ligases containing one or more regulator of chromosome condensation 1 (RCC1)-like domains in their amino-terminal domain form a HERC subgroup [2]. HERC1 and HERC2 are the largest HECT ligases, having molecular weights exceeding 500 kDa, and constitute the large HERC protein subfamily [3]. By contrast, HERC3 to HERC6 have molecular weights around 100–120 kDa and constitute the small HERC protein subfamily. Despite the structural similarity between large and small HERC proteins (Figure 1), they are evolutionarily very distant. In fact, they are the result of convergence phenomena rather than being phylogenetic paralogs [3,4,5]. Moreover, the small HERC proteins HERC5 and HERC6 may also function as ISG15 E3 ligases [6,7].

## 2. The Role of HERCs in Cancer

HERCs play roles in a wide range of cellular functions, including neurodevelopment, cell response to replication stress and DNA damage, cell proliferation, cell migration, and immune responses. As such, mutations in HERCs are associated with severe pathologies [3,6,8], with a notable impact in cancer. An extensive list of the different cancers associated with the specific large and small HERCs is provided in Table 1.

Mutations in large HERCs have been found in leukemia [10,11,12,13] and breast cancer [16,17]. Frameshift mutations in *HERC2* have been found in both gastric and colorectal carcinomas with microsatellite instability [31]. The *HERC2* locus has also been associated with both cutaneous melanoma and uveal melanoma, whereas the *HERC1* locus has been found to be mutated in non-melanoma skin cancer [20,21,25,27]. Higher expression levels of HERCs are associated with better patient prognosis in kidney, head and neck, and pancreatic cancers when *HERC1* expression levels are elevated, and in patients with renal cancer when *HERC2* expression levels are elevated [50]. By contrast, the expression levels of HERC2 have been found to negatively correlate with patient survival in non-small-cell lung cancer [28]. In osteosarcoma, upregulation of the HERC2-binding protein SOX18 enhances cell proliferation, and it correlates with a reduction in both large and small HERC mRNA levels (Table 1) [18].

Several studies using gene expression analyses of different tumor tissues have showed differential expression patterns of small HERCs between healthy and tumor samples as well as between tumors at different stages. This raises the possibility of using small HERCs as diagnostic or prognostic biomarkers for different cancer types. For instance, *HERC4* and *HERC5* expressions have been shown to correlate with tumor progression in breast cancer [37,45] and have been implicated in both lung [34,35,36,44] and hepatocellular [39,46,47,51] carcinomas. In addition, high levels of *HERC3* expression correlate with poor prognosis in glioblastomas [32], while *HERC5* appears to be differentially expressed and associated with progression in acute myeloid leukemia (Table 1) [42].

## 3. Signaling Pathways Regulated by HERCs

Gene expression and survival studies suggest that HERCs have different roles in cancer. Gaining a better understanding of HERCs can be facilitated by understanding the intracellular signaling pathways where HERCs operate. HERCs appear to have bivalent functions, acting as either oncogenes or as tumor suppressors depending on the tumor type. In turn, this complicates research into the relevant signaling pathways where HERCs participate. In recent years, several studies have contributed to our understanding of the tumor suppressor function of large HERCs, with three major regulatory mechanisms emerging: regulation of genomic stability, regulation of p53 transcriptional programming, and regulation of mitogen-activated protein kinase (MAPK) signaling.

### 3.1. Regulation of Genomic Stability

**Figure 2:**
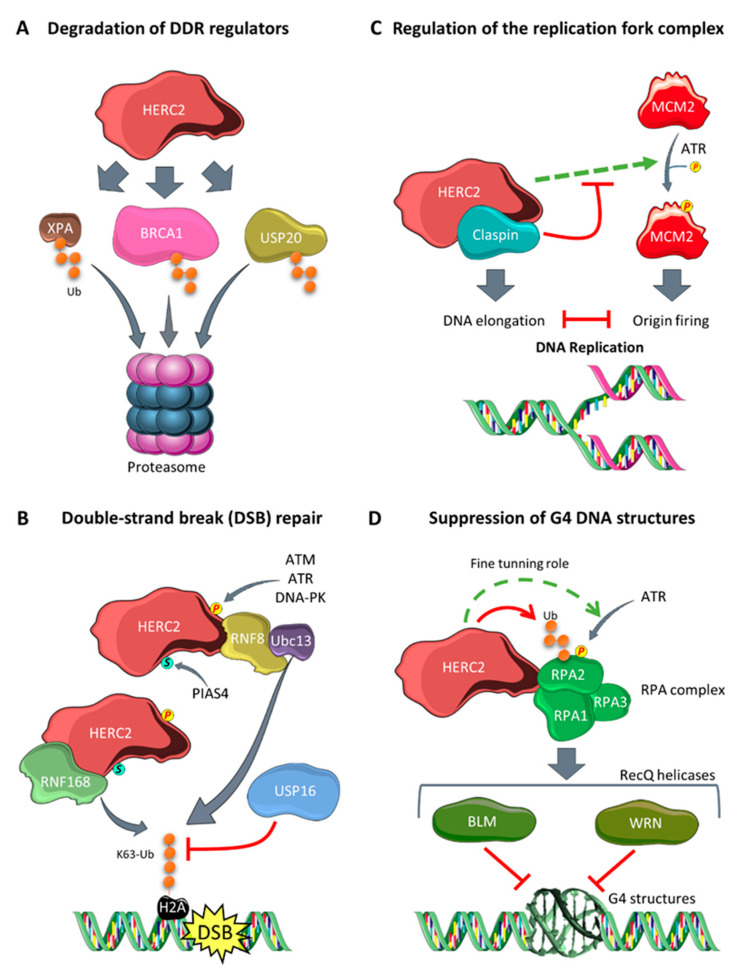
Involvement of HERC2 in the regulation of genomic stability: (**A**) HERC2 catalyzes the polyubiquitylation of different DNA damage response (DDR) regulators, such as XPA, BRCA1, and USP20, targeting them for proteasomal degradation; (**B**) upon double-strand break in the DNA, HERC2 is phosphorylated (by ATM, ATR, or DNA-PK) and SUMOylated (by PIAS4). These posttranslational modifications allow HERC2 to bind to RNF8, promoting the specific assembly of RNF8 with the E2 enzyme Ubc13. This allows the formation of K63 polyubiquitin chains in H2A-type histones flanking the double-strand break site. HERC2 interacts with and stabilizes RNF168, and this amplifies the ubiquitin chain formation in histones. By contrast, USP16 levels increase in a HERC2-dependent manner and negatively regulate H2A histone ubiquitylation to fine-tune chain formation; (**C**) HERC2 is present in the replication fork complex that regulates the balance between DNA elongation and origin firing. HERC2 facilitates ATR-dependent MCM2 phosphorylation, which enhances origin firing, and this is inhibited by Claspin, another HERC2-interacting protein. (**D**) G4 structures cause replication stress, and this leads to HERC2 promoting RPA2 phosphorylation via ATR. Then, HERC2 polyubiquitylates the phosphorylated form of RPA2, targeting it for proteasomal degradation, and thereby fine-tuning the total levels of phospho-RPA2. This mechanism is essential for assembling the BLM and WRN RecQ helicases to the RPA complex and for its later role in suppressing G4 structures.

Genomic instability is a hallmark of cancer that is observed in the early stages of tumorigenesis [52,53]. HERC2 participates in the modulation of genomic stability through degradation of key regulators of DNA repair pathways. Xeroderma pigmentosa A (XPA) is critical to the nucleotide excision repair machinery. It is ubiquitylated by HERC2, which targets it for proteasomal degradation in a circadian-dependent manner (Figure 2A) [54]. The regulation of this process depends on the phosphorylation state of XPA. Its phosphorylation by serine/threonine-protein kinase ATR prevents ubiquitylation by HERC2, and its dephosphorylation by protein phosphatase 1D (also known as WIP1) enhances its ubiquitylation by HERC2 [54,55].

Another HERC2 degradation target is breast cancer suppressor 1 (BRCA1) (Figure 2A) [29]. BRCA1 maintains genome stability by repairing double-strand breaks [56]. The HERC2-dependent degradation of BRCA1 is regulated by the binding of different protein interactors to HERC2 or BRCA1; for example, BARD1 binds BRCA1, forming a heterodimer that prevents BRCA1 ubiquitylation by HERC2. Similarly, TUSC4 binds to HERC2 and prevents BRCA1 degradation, with its knockdown consistently shown to enhance BRCA1 degradation through the proteasome pathway [29,30].

Further, also concerning the response to DNA damage, HERC2 is required for the retention of DNA repair factors such as 53BP1, RAP80, RNF168, and BRCA1 at sites of DNA damage in response to double-strand breaks [57]. Upon double-strand breaks, HERC2 is phosphorylated on T4827 by serine-protein kinase ATM or other DNA damage-related kinases like ATR or DNA-PK. In addition, it can be SUMOylated in its ZZ domain by the E3 SUMO ligase PIAS4 [58]. These posttranslational modifications allow the binding of HERC2 to RNF8, another ubiquitin E3 ligase involved in the DNA damage response. In these conditions, HERC2 promotes the specific assembly of RNF8 with one of its E2 enzymes, Ubc13, which catalyzes the formation of K63 polyubiquitin chains formed in H2A-type histones flanking the double-strand break site. HERC2 therefore mediates the specificity of the interaction between the E2 enzyme Ubc13 and the E3 ligase RNF8, safeguarding the formation of K63 ubiquitin chains that are essential for recruiting repair factors to the damaged chromosomes. In parallel, HERC2 can interact with, and stabilize, the E3 ligase RNF168 that is responsible for amplifying the ubiquitin chain formation initiated in histones by RNF8 [57,58]. Levels of the deubiquitinase USP16 then increase in a HERC2-dependent manner during DNA damage, and this negatively regulates H2A histone ubiquitylation to fine-tune polyubiquitin chain formation. Ultimately, USP16 triggers the ubiquitylation signal termination once the damage is repaired. These findings suggest that HERC2 has a central role in regulating and fine-tuning the DNA damage response pathway (Figure 2B) [59].

HERC2 also regulates the levels of Claspin, a protein essential for both G_2_-M checkpoint activation and replication fork stability. HERC2 interacts with Claspin in the presence of BRCA1 [60]. Upon DNA damage, ATR phosphorylates USP20, a deubiquitinating enzyme which is a degradation target of HERC2 (Figure 2A). USP20 detaches from HERC2, leading to its stabilization, and in turn deubiquitylates and stabilizes Claspin levels. The Claspin can then interact with the serine/threonine-protein kinase CHK1, promoting ATR-dependent phosphorylation, checkpoint activation, and genome stability [61,62]. Moreover, HERC2 has been described as a component of the DNA replication fork complex that regulates origin firing and fork progression. During DNA replication, origin firing and elongation must be correctly balanced. Claspin and CHK1 enhance elongation but restrict origin firing. When faced with replication stress that impairs DNA elongation, HERC2 facilitates the phosphorylation and activation of the DNA replication licensing factor MCM2 (Figure 2C). Thus, origin firing is upregulated as a compensatory mechanism [60]. Dysregulation of these replication mechanisms can lead to DNA damage and genome instability [63].

Some guanine-rich DNA sequences can fold into a secondary structure known as G-quadruplex (G4). These structures are involved in transcriptional initiation and termination, replication initiation, and telomere maintenance. However, the deregulation of G4 can cause fork stalling during DNA replication that promotes DNA damage and genome instability. HERC2 interacts with replication protein A (RPA), a heterotrimeric complex formed by RPA1, RPA2, and RPA3 and that binds to single-stranded DNA and protects it from spontaneous annealing and G4 formation. RPA assembly to the RecQ DNA helicases BLM and WRN is essential for suppressing the formation of G4 DNA structures. Under conditions of replication stress, ATR phosphorylates RPA2 at Ser33 in an HERC2-dependent mechanism. Then, HERC2 ubiquitylates the phosphorylated form of RPA2, targeting it to proteasomal degradation [64]. Although the specific effects of RPA2 phosphorylation and its later degradation by HERC2 are not fully understood, both mechanisms are essential for HERC2 to function correctly in suppressing G4 structures. HERC2 may fine-tune the levels of phosphorylated RPA2 by promoting its ATR-dependent phosphorylation and targeting it for proteasomal degradation through polyubiquitylation. This precise balance might be needed for the correct assembly of the BLM and WRN helicases to the RPA complex, as well as for the subsequent suppression of G4 structures and the maintenance of genomic stability (Figure 2D) [65].

HERC1 has also been associated with the DNA damage response through its regulation of MSH2, which forms heterodimers with MSH6 and MSH3. Both these dimers are responsible for the DNA mismatch repair mechanism, and loss of function impairs DNA repair. Depleting HERC1 has been linked to reduced protein MSH2 levels in human leukemia cells, leading to increased genomic instability and chemotherapy resistance [11].

### 3.2. Regulation of p53 Transcriptional Programming

**Figure 3:**
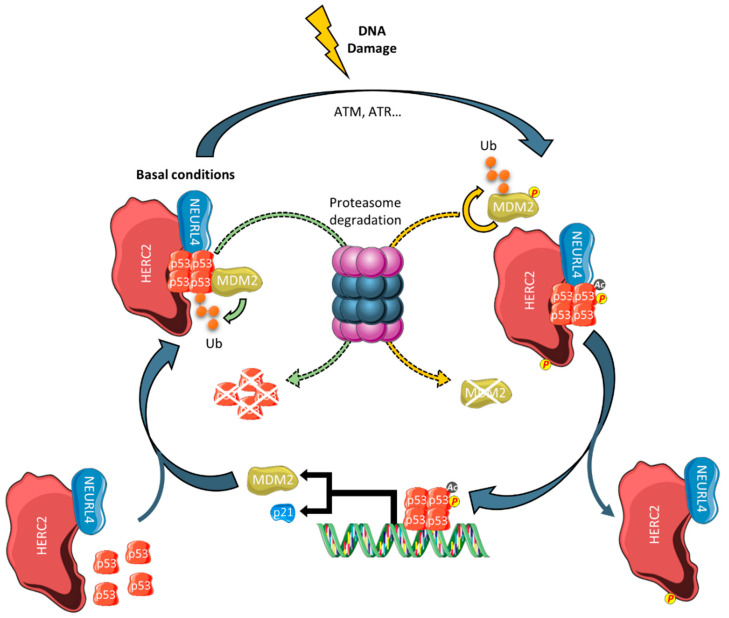
Regulation of the p53/MDM2 pathway by HERC2. In basal conditions, HERC2 and NEURL4 regulate p53 transcriptional activity by promoting its oligomerization. MDM2 maintains low levels of p53 by targeting it for proteasomal degradation. Upon DNA damage, associated kinases phosphorylate MDM2 and HERC2. In addition, p53 is activated by acetylation and phosphorylation. The phosphorylated MDM2 is detached from the complex and is autopolyubiquitylated and subsequently degraded. Activated p53 binds to the promoters of its target genes, such as p21 or MDM2, and a negative feedback loop is established in the latter case.

Large HERCs may also function as tumor suppressors by regulating gene expression. For example, HERC2 controls the transcriptional activity of the tumor suppressor protein p53 [66]. Tetramerization of p53 is a key step in its activation, and this oligomerization process is modulated by a complex formed by NEURL4 and HERC2 [67]. Recently, it has been reported that MDM2 is present in this complex. In non-stressed cells, this ubiquitin E3 ligase promotes lower levels of p53 by targeting it for proteasome degradation. Upon DNA damage induced by bleomycin, MDM2 is dissociated from the p53/HERC2/NEURL4 complex, and phosphorylation and acetylation of oligomeric p53 increases. Activated p53 then binds to the p53 response elements in the promoters of its target genes. *MDM2* is one such gene whose promoter competes with HERC2 for binding of oligomeric, phosphorylated, and acetylated p53. As its gene expression increases MDM2 protein levels, these can bind p53 and restart the regulatory loop (Figure 3) [68]. These findings highlight the importance of HERC2 in regulating the transcriptional program of the tumor suppressor protein p53.

### 3.3. Regulation of MAPK Signaling

MAPKs are organized in three-tiered cascades regulated by phosphorylation: MAPKK kinases (MAPKKKs) are serine/threonine-protein kinases that phosphorylate and activate MAPKKs, which in turn phosphorylate the MAPKs that mediate the cellular response by phosphorylating effector proteins. This signaling has therefore been shown to be key for cell differentiation, growth, proliferation, and survival. The classic MAPK cascades include Jun N-terminal kinase (JNK), p38, and extracellular signal-regulated protein kinase (ERK) pathways. Dysregulation of these cascades has been linked extensively to oncogenesis [69,70,71].

**Figure 4:**
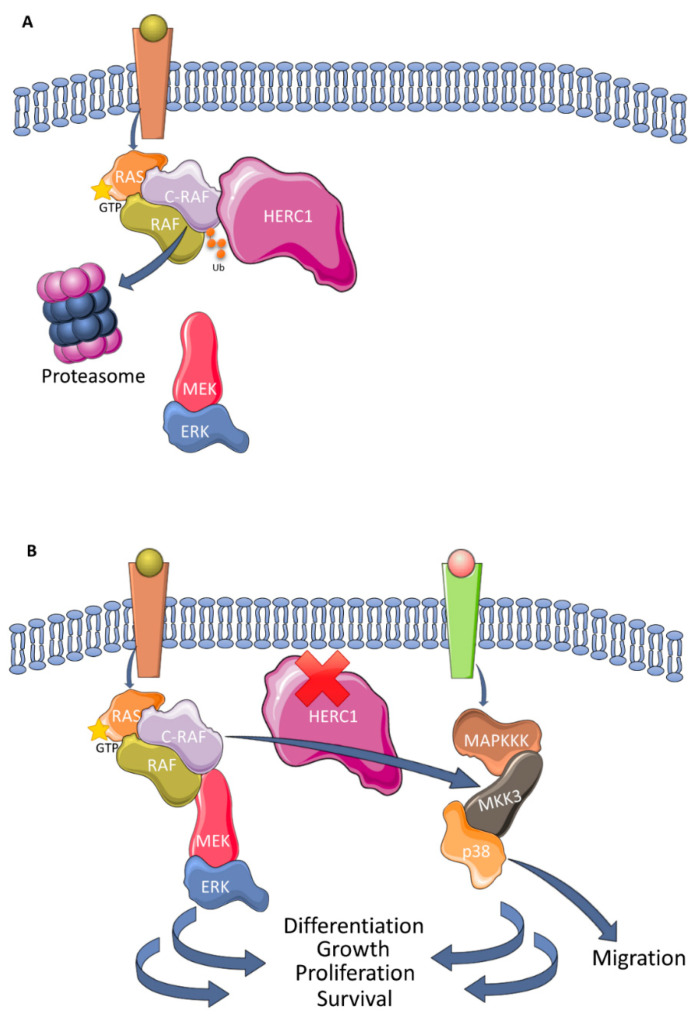
Regulation of MAPK signaling pathways by HERC1: (**A**) HERC1 polyubiquitylates C-RAF with a lysine 48-linked chain that causes its degradation by the proteasome and downregulates ERK activity; (**B**) in the absence of HERC1, the levels of C-RAF increase and upregulate the RAF/MEK/ERK signaling pathway. The levels of MKK3 also increase, which in turn phosphorylates and activates p38. In summary, therefore, the absence of HERC1 may enhance cell responses regulated by these MAPKs, including the migration, differentiation, growth, proliferation, and survival of cells.

HERC1 and HERC2 each have roles in MAPK signaling. For example, HERC2 has been reported to be part of a high-molecular weight complex formed by UBE3A, NEURL4, and MAPK6/ERK3 [72]. Some cellular functions that might be regulated by this complex involve transcription, protein translation, formation and transport of vesicles, as well as metabolism [73]. To date, the precise molecular mechanism remains to be elucidated, necessitating further research. By contrast, our knowledge of the regulation of MAPK by HERC1 has evolved and grown in recent years. HERC1 controls cell proliferation through the regulation of ERK signaling by targeting the proto-oncogene C-RAF, an MAPKKK, for proteasomal degradation (Figure 4A) [74]. The HERC1 ligase also regulates the p38 pathway and cell migration. Research indicates that HERC1 knockdown increases p38 activity by increasing the levels of MKK3, an MAPKK of the p38 pathway, in a manner dependent on C-RAF. Thus, HERC1 regulates a previously unknown crosstalk between these two MAPK signaling pathways [75]. In this context, it is postulated that HERC1 functions as a tumor suppressor protein, given that its depletion leads to the dysregulation of two essential cascades for tumorigenesis (Figure 4B).

## 4. Perspective and Therapeutic Implications

The ubiquitin proteasome system has been targeted for cancer therapy through proteasome inhibitors such as bortezomib, and these are achieving promising results in ongoing clinical trials. However, given that the treatments lack specificity, they can induce clinically relevant side effects [76]. Hence, the possibility of targeting E3 ligases, which display high substrate specificity, may help achieve more specific and efficient therapies. Given the possible tumor suppressor role of large HERCs in some cancers, a potential therapeutic approach would be to rely on enhancing their tumor suppressor activities. Following this theory, both HERC1 and HERC2 could be potential drug candidates. On the one hand, promoting the capacity of HERC2 to oligomerize p53 using the CPH domain of HERC2 [66] could boost the p53 transcriptional program and potentially alleviate tumor progression in tumors with non-mutated p53. On the other hand, a growing body of evidence suggests that targeting C-RAF could be a promising therapeutic strategy based on its efficiency in K-Ras and Trp53 mutant mice models of lung [77] and pancreatic ductal [78] adenocarcinomas. Enhancing HERC1 ubiquitin ligase activity in this context could be an interesting approach to stop tumor progression in MAPK-dependent cancers. These approaches could also be combined with other therapeutic strategies that target the inhibition of MDM2 with Nutlins in the case of HERC2, and epidermal growth factor receptor activity with monoclonal antibodies or tyrosine kinase inhibitors in the case of HERC1.

Given the role of large HERCs in DNA damage response, inhibitors for these proteins could be designed to exploit synthetic lethality as a therapeutic strategy in some cancers. Deficiencies in the DNA repair mechanisms of tumor cells can be exploited by inhibiting the remnant mechanisms and triggering tumor cell death [79]. Many genes involved in DNA repair mechanisms are mutated in tumors [80]. Synthetic lethality also benefits from the fact that cancer cells present higher replication stress due to their aberrant proliferation [81]. The viability of synthetic lethality strategies has been demonstrated with the use of poly [ADP-ribose] polymerase (PARP) inhibitors, since tumor cells harboring BRCA1/2 mutations are 1000 times more sensitive to PARP inhibition than wild-type counterparts [82]. Given this evidence, large HERC inhibitors, especially those for HERC2, could eventually be of use in this setting.

## 5. Conclusions

HERCs can function as either oncogenes or tumor suppressors depending on the cancer type. This bivalent activity hinders the study and understanding of the regulatory mechanisms where they operate. Although important cellular processes such as genome stability, gene expression, and MAPK signaling are regulated by HERCs proteins, future in vivo and in vitro studies are required to elucidate the molecular mechanisms where HERCs participate and to identify new substrates specific for each cancer type. Screenings for activator and inhibitor molecules for their ubiquitin ligase activities could also be useful when developing novel therapeutic strategies.

## Figures and Tables

**Table 1:** Cancers associated with HERCs and related molecular mechanisms.

Genes	Associated Cancers	Related Molecular Mechanisms	Reference
***HERC1***	Acute promyelocytic leukemia	*HERC1-PML* genomic fusion	[9]
	Acute Myeloid Leukemia	*HERC1* mutations	[10]
	Acute lymphoblastic leukemia	Decreased MSH2 protein levels and *HERC1* deletions	[11]
	Adult T-cell acute lymphoblastic leukemia	*HERC1* mutations	[12]
	T-cell prolymphocytic leukemia	*HERC1* mutations	[13]
	Non-melanoma skin cancer	Enhanced BAK protein degradation	[14]
	Pulmonary sclerosing pneumocytoma	*HERC1* mutations	[15]
	Invasive lobular breast cancer	*HERC1* mutations	[16]
	Metastatic triple-negative breast cancer	*HERC1* mutations	[17]
	Sporadic colorectal cancer	Decreased MSH2 protein levels and *HERC1* deletions	[11]
	Osteosarcoma	Negative correlation of *SOX18* overexpression and *HERC1* mRNA levels	[18]
***HERC2***	Pheochromocytoma and paraganglioma	*HERC2* mutations	[19]
	T-cell prolymphocytic leukemia	*HERC2* mutations	[13]
	Cutaneous melanoma	SNPs in *HERC2* gene increase susceptibility	[20,21,22,23]
		Gene-gene interactions between *HERC2* gene and *IL31RA* and *DDX4* genes	[24]
		Epistatic effects between *HERC2* and *VDR* genes	[25]
	Cutaneous squamous cell carcinoma	SNPs in *HERC2* gene impact on time to develop the tumor in organ transplant recipients	[26]
	Uveal melanoma	SNPs in *HERC2* gene increase susceptibility	[27]
	Non-small-cell lung cancer	Worse prognosis in patients expressing high *HERC2* mRNA levels	[28]
	Breast cancer	Enhanced BRCA1 degradation	[29,30]
	Gastric and colorectal carcinomas	*HERC2* mutations	[31]
	Osteosarcoma	Negative correlation of *SOX18* overexpression and *HERC2* mRNA levels	[18]
***HERC3***	Glioblastoma	Degradation of SMAD7 and activation of the TGFβ signaling	[32]
	Gastric and colorectal carcinomas	*HERC3* mutations	[31]
	Osteosarcoma	Negative correlation of *SOX18* overexpression and *HERC3* mRNA levels	[18]
***HERC4***	Multiple myeloma	Decreased c-Maf degradation	[33]
	Lung cancer	*HERC4* overexpression	[34]
	Non-small cell lung cancer	Increased Smo protein stability and Hh pathway activation	[35,36]
	Breast cancer	*HERC4* upregulation	[37]
		Decreased expression of miRNAs targeting *HERC4* expression and enhanced LATS1 degradation	[38]
	Hepatocellular carcinoma	*HERC4* overexpression	[39]
	Osteosarcoma	Negative correlation of *SOX18* overexpression and *HERC4* mRNA levels	[18]
***HERC5***	Pediatric germ cell tumors	Chromosome copy number variations (CNVs) at a region encompassing *HERC5* gene	[40]
	Glioblastoma	*HERC5* upregulation	[41]
	Acute myeloid leukemia	*HERC5* downregulation	[42]
	Oropharyngeal cancer	*HERC5* gene expression is associated with overall survival	[43]
	Non-small cell lung cancer	*HERC5* promoter hypermethylation	[44]
	Breast cancer	*HERC5* upregulation	[45]
	Hepatocellular carcinoma	Negative correlation of *CCL20* overexpression and *HERC5* mRNA levels	[46]
		Reduced p53, p21 and Bax/Bcl-2 pathway activation	[47]
	Ovarian cancer	*HERC5* upregulation is associated with drug resistance	[48,49]
	Osteosarcoma	Negative correlation of *SOX18* overexpression and *HERC5* mRNA levels	[18]
***HERC6***	Osteosarcoma	Negative correlation of *SOX18* overexpression and *HERC6* mRNA levels	[18]
